# Timing of implantation and extraction in aesthetically sensitive anterior tooth region, part 1: Clinical case reports

**DOI:** 10.1097/MD.0000000000042296

**Published:** 2025-04-25

**Authors:** Thorsten Koszlat

**Affiliations:** a Implantological and Surgical Dental Practice Frankfurt, TreDento, Frankfurt am Main, Germany.

**Keywords:** aesthetic implantology, anterior tooth implantation, immediate implantation, tooth infection

## Abstract

**Rationale:**

Immediate implantation after anterior tooth trauma is the safest method for completely preserving the alveolar structures. However, based on evidence, most anterior tooth trauma occurs in childhood when implantation is not possible owing to growth in the jaw area.

**Patient concerns:**

In adulthood, long-term effects can occur with cyst formation or chronic infections in traumatized teeth. The loss of an anterior tooth leads to the loss of alveolar structures and aesthetic limitations due to recession, scar tissue, or surgical augmentation procedures.

**Diagnoses:**

Patient 1 was a 21-year-old woman with an extensive radicular cyst following anterior tooth trauma in early childhood. She had incomplete root growth and an open apex. Patient 2 was a 24-year-old woman with a history of anterior tooth trauma in 11 and 21 during adolescence. She also had fistulas in regions 11 and 21.

**Interventions:**

In both patients, optimally timed implantation and minimally invasive surgery resulted in tissue preservation without additional augmentation procedures.

**Outcomes:**

This case series highlights varying clinical presentations of childhood anterior tooth trauma and their long-term effects in adulthood.

**Lessons:**

By optimizing the timing of extraction and implantation, alveolar structures were fully preserved without the need for additional augmentation procedures.

## 1. Introduction

**Figure 1.**
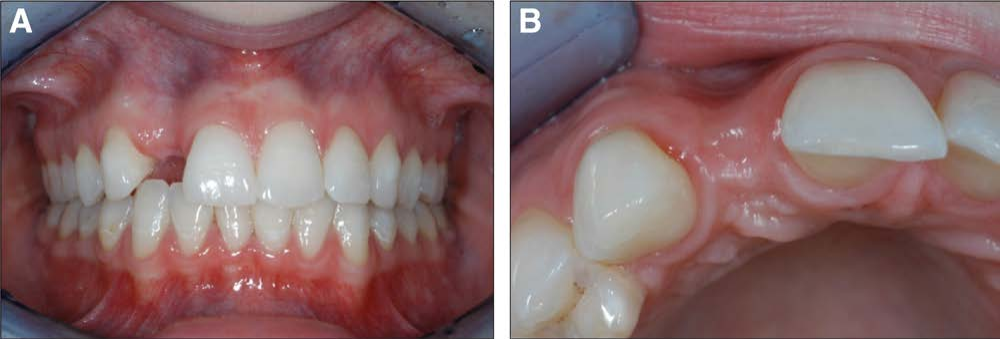
(A, B) Twelve months after anterior tooth trauma and several months of healing, the vestibular bone lamella was lost, resulting in a concave bone shape.

The management and timing of implantation and tooth extraction directly influence pink-white aesthetics, particularly in the aesthetically sensitive anterior tooth region.^[1-7]^ Preserving the natural alveolar ridge and mucous membrane while ensuring their physiological progression is a key goal in achieving a harmonious smile, especially in aesthetic implantology.^[3,4,8-19]^ In literature, immediate implantation, which occurs directly after the extraction of an anterior tooth, is distinguished from 2-stage implantation technique. Early 2-stage implantation is performed after soft tissue healing (within 6 weeks post-extraction), while late 2-stage implantation, occurs after complete soft tissue healing, or just prior to bony healing of the extraction socket (approximately 5–6 months after extraction).^[12,19-22]^ When determining the optimal time for implantation, 2 factors play a decisive role: the progressive resorption of the thin vestibular alveolar wall following extraction of an anterior tooth and the inflammation-free osseointegration of the implant, which is crucial for achieving a natural-looking pink-white aesthetic (Fig. 1).

Resorption due to insufficient loading of the alveolar bone often results in loss of the thin vestibular alveolar wall, frequently causing concave invaginations of the alveolar ridge in the region where implanted is placed. This physiological bone loss process in the aesthetically important anterior tooth area is most pronounced in the first 3 to 6 months and the alveolar bone loss can be up to 29% to 63% in the horizontal dimension and up to 11% to 22% in the vertical dimension.^[10,23,24]^

This condition directly affects subsequent surgical procedures and aesthetic outcomes, aiming to achieve a natural and harmonious aesthetic profile with an optimal pink-white balance of the implant prosthesis that aligns well with adjacent teeth. Additional surgical interventions for soft tissue or bone augmentation may be required, particularly if the vestibular alveolar wall shows significant bone deficiency. However, these augmentation procedures significantly increase the number of surgical interventions, which introduces additional risks beyond the medical need for implantation.

These risks include the formation of postoperative scar tissue, aesthetic compromise in the mucogingival region of the anterior teeth, and the possibility of recession around adjacent natural teeth. Such complications often arise from extensive manipulation of mucogingival soft tissue, vertical releasing incisions, and the creation of mucosal or mucoperiosteal flaps, which can lead to displacement of the natural mucogingival line in the aesthetic zone.

In cases of autologous bone augmentation, there is also a risk of granulomatous resorption of bone grafts or failure of cortical bone healing, which can result in further hard and soft tissue deficits in the anterior tooth region. When xenogenic or synthetic bone graft materials are used, there is a high risk of failure owing to postoperative infections. In such cases, the non-autologous graft may need to be removed during a second surgical procedure, causing additional trauma. Loss of both hard and soft tissues due to these complications can severely limit aesthetic outcomes in the mucogingival anterior tooth region or lead to complete failure of implantation.

In general, unnecessary surgical interventions particularly in the aesthetically sensitive anterior tooth region, does not improve the initial aesthetic outcome after implantation. Several case studies have demonstrated that with proper timing of tooth extraction followed by implantation, additional augmentation techniques involving flap creation can be avoided. This makes the overall aesthetic and implantological outcomes more predictable and manageable for surgeons.

Due to the physiological process of bone resorption and the surgical risks associated with additional augmentation procedures, delaying implantation prolongs alveolar bone healing. Therefore, early implantation is recommended to preserve the alveolar bone and maintain anterior tooth aesthetics. Immediate implantation meets these criteria by preserving the alveolar bone and mucogingival soft tissue structures. However, in cases of acute alveolar infection and pronounced local pathologies, the risk of wound infection or bacterial contamination can be minimized by delaying implantation time from immediate to early.^[21,25]^

Even with early implantation (6–8 weeks post-extraction), soft tissue or bone augmentation may still be necessary due to the resorption of the thin vestibular alveolar wall. The following case reports show that early implantation may not be a viable alternative, as apical bone deficits could lead to irreversible vestibular bone collapse. By optimizing the timing of implantation, the existing acute tooth infection was treated first, and immediate implantation was performed once complete apical bone regeneration was achieved.

In a systematic review from 2011, which included only prospective studies with a follow-up of at least 12 months, a very high 2-year survival rate of 98% was reported for immediate implantation.^[26]^ Another systematic review analyzing 34 studies (956 implants, 29 losses) demonstrated a 1-year survival rate of 97% for single immediate implantations in the aesthetic zone.^[15]^

## 2. Case report 1

Extensive radicular cyst in a 21-year-old female patient following anterior tooth trauma in early childhood with incomplete root growth and an open apex.

**Figure 2.**
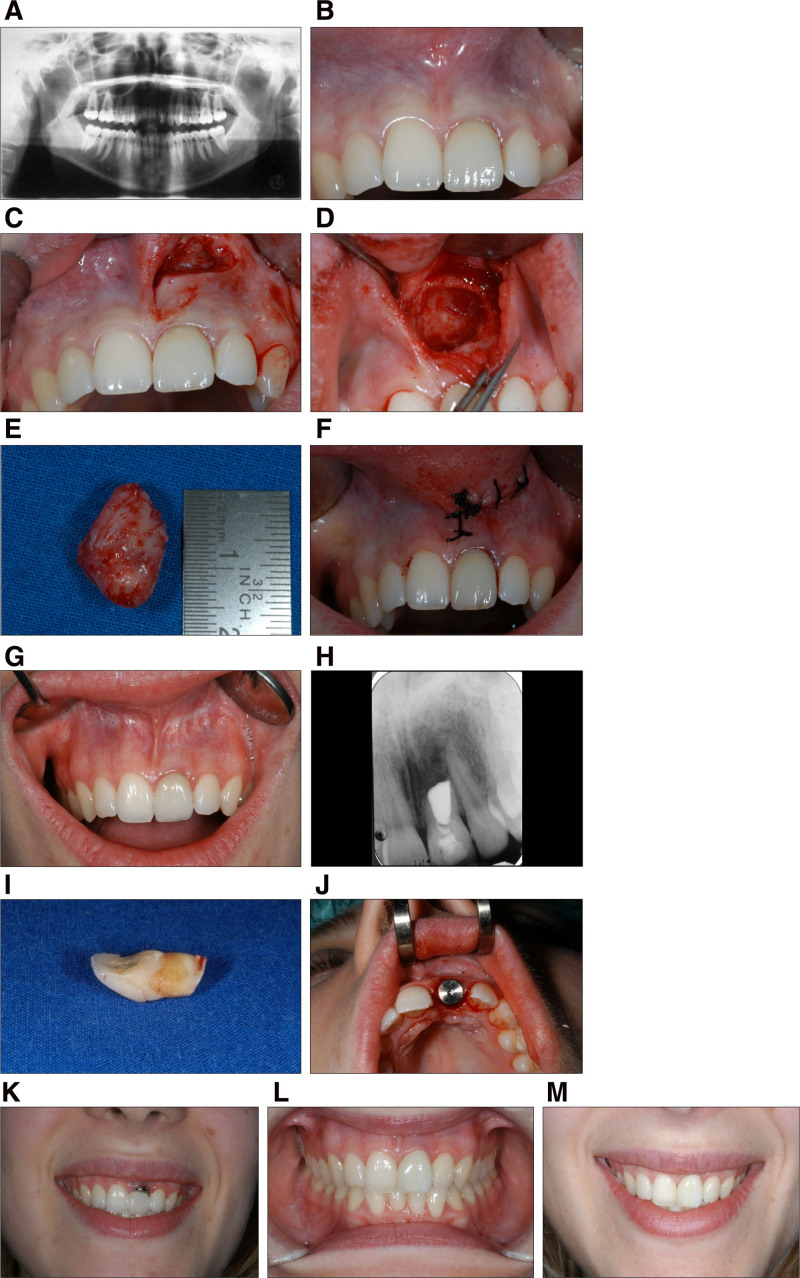
(A, B) Radiological and clinical findings after abscess healing. (C–F) Angled incision in the vestibule and cystectomy of the extensive radicular cyst, along with root apex resection and retrograde filling, for the temporary retention of tooth 21 until complete ossification of the cyst lumen. (G, H) After a 12-mo healing phase of the cyst lumen, radiographic evaluation confirmed sufficient bone regeneration, allowing for the extraction of tooth 21 and immediate implantation with transgingival healing. All anatomical structures of the aesthetic zone were completely preserved, as seen in the initial findings. (I, J) Immediate implantation was performed after the tissue-sparing extraction of the remaining tooth 21. (K) The patient’s high smile line, shown with the gingival former, highlights the possible aesthetic concerns caused by the loss of the scalloped red-white mucogingival aesthetics. (L, M) After complete osseointegration of implant 21, the tooth was restored using a ceramic abutment and a zirconium oxide ceramic crown.

The patient presented with an acute abscess on the upper lip in the fossa canina region. An orthopantomogram (Fig. 2A) revealed an extensive radiolucent, cystic lesion extending from tooth 21 to the region of tooth 22 and reaching the floor of the nasal cavity.

Apart from the abscess, the patient had no prior symptoms related to tooth 21. No swelling, pain, or abnormal tooth mobility was observed. The diagnosis was a radicular cyst, a chronic odontogenic cyst resulting from persistent inflammation at the root apex, originating from the epithelial rests of Malassez, which remain in the apical region after tooth development. Over time, the cyst expands osmotically, progressively resorbing bone and potentially displacing adjacent tooth roots. Radicular cysts are usually an incidental radiological finding, since they are typically asymptomatic unless complicated by an acute infection.

The patient had a high smile line. Owing to aesthetic and color deviations, tooth 21 was treated with a veneer by a previous practitioner. The initial aesthetic situation was visually perfect (Fig. 2B); however, immediate implantation was contraindicated because of the cystic space-occupying lesion. The cyst had to be removed first following the acute infection (Fig. 2C–F). Tooth 21 was not worthy of preservation and extensive soft tissue surgery with sulcular and vertical releasing incisions for extraction and simultaneous cystectomy, would have resulted insignificant bone collapse. Even with extensive bone and soft tissue augmentation, restoring aesthetics surgically would have been extremely challenging.

The main aim of the initial surgical procedure was to preserve the natural aesthetic alveolar structures for future implantation in region 21. Therefore, the initial surgical procedure was to remove the source of the acute infection causing the upper lip abscess while ensuring the preservation of the aesthetic mucogingival region.

Surgically, a flap was created outside the aesthetic mucogingival zone in the area of the movable vestibule with a reverse-angled incision near the nasal floor and frenulum of the lip. A cystectomy was then performed in the apical region of tooth 21 (Fig. 2C–F).

After simultaneous orthograde and retrograde access to the root canal of tooth 21 during the apicoectomy and cystectomy, the root canal could be completely disinfected and cleaned under direct vision. This enabled complete smoothening of the root apex and placement of a large retrograde filling with IRM© (Intermediate Restorative Material, Dentsply Sirona, DentsplayDeTrey GmbH, Germany).

Although tooth 21 was non-restorable, it was temporarily preserved with retrograde endodontic filling with IRM to stabilize the vestibular alveolar bone during cyst cavity healing (Fig. 2G, H) and to prevent reinfection of the bone.

During the first 12 weeks postoperatively, the patient was instructed to avoid biting into hard foods with her front teeth to prevent overloading tooth 21 and ensure stability until complete bone healing. Soft tissue and subsequent bone healing proceeded without irritation or complications under initial perioperative antibiotics.

In the first phase of bone healing, various proteins contribute to the formation of a coagulum, which stabilizes the wound and its edges through mechanical bonding. As healing progresses, woven bone begins to form.

During the nonspecific cellular phase, platelets within the blood clot release signaling molecules that act on cells in the soft tissue, the wound margins, and circulating blood cells. Growth factors such as Transforming Growth Factor Beta, Insulin Like Growth Factor, and Platelet Derived Growth Factor promote the migration of proliferating cells and macrophages into the blood clot, facilitating phagocytosis of the blood clot. Concurrently, new blood vessels are formed and a collagen framework develops, and granulation tissue emerges as the wound contracts.

In the bone-specific cellular phase, bone stem cells migrate into the defect through the exposed bone marrow cavity, where they differentiate into tissue-specific cells. Bone morphogenic protein stimulates the differentiation of stem cells into osteoprogenitor cells, which are precursors to osteoblasts. Complete bone healing depends on the size of the defect and typically takes 6 to 12 months.

Following radiographic confirmation of complete bony healing of the cyst lumen, tooth 21 was extracted (Fig. 2I), and immediate implantation was performed12 months after cystectomy (Fig. 2J).

The high smile line posed an additional challenge, as any irregularity in the natural contour of the teeth and gums would permanently disturb the aesthetics of the natural smile line, as demonstrated by the example of the temporary gingival abutment (Fig. 2K).

Due to the optimal timing of extraction and implantation, along with minimally invasive surgical techniques, the natural mucogingival soft tissue architecture was fully preserved without the need for additional augmentation procedures (Fig. 2L, M).

**Ethical Review Statement**: Ethical review and approval were waived because the images are available for use in a scientific paper. It is a case report, and the clinical images serve to explain the evidence.

**Informed Consent Statement:** A declaration of consent for the creation of intraoral photo documentation throughout the treatment for scientific purposes was obtained verbally and in writing from all subjects involved in the study at the beginning of the treatment.

## 3. Case report 2

Fistulas in regions 11 and 21 in a 24-year-old female patient with a history of anterior tooth trauma to teeth 11 and 21 during adolescence.

**Figure 3.**
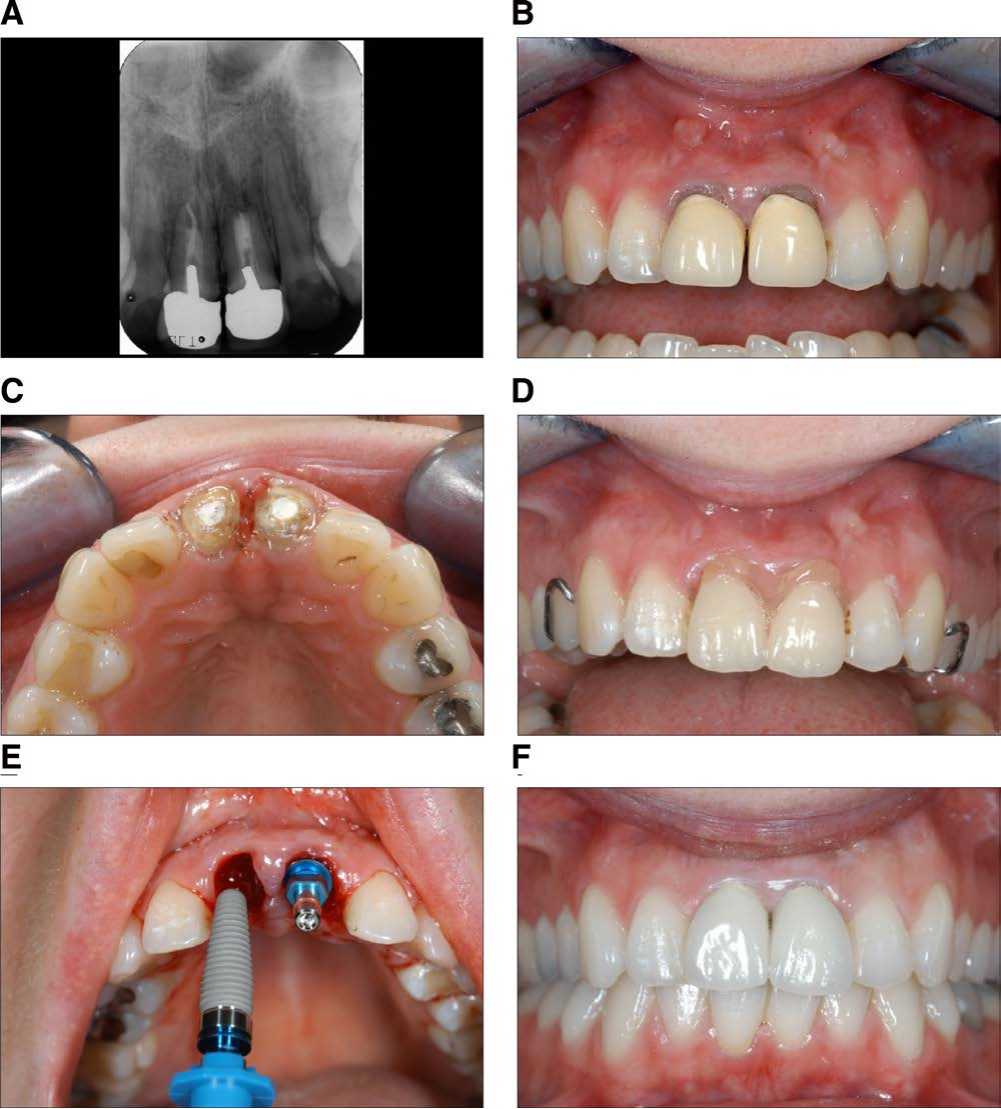
(A, B) Initial radiological and clinical findings. (C, D) Temporary filling of the anterior tooth spacing with an interim prosthesis. (E, F) Final prosthetic restoration of the implants with cemented full-shell ceramic crowns.

Radiological examination revealed prior endodontic and prosthetic treatment, including post-and-crown restorations and multiple surgical root apex resections performed to preserve the teeth into adulthood (Fig. 3A). The patient presented with recurrent apical periodontitis and chronic fistulas in the vestibular regions of teeth 11 and 21 (Fig. 3B). Despite the presence of vestibular fistulas, teeth 11 and 21 were clinically stable, showing no pathological mobility or pain. A periodontal-endodontic lesion was excluded. Due to the chronic inflammatory reaction and bacterial contamination of the periapical region, immediate implantation was contraindicated.

The bacterial flora in odontogenic periapical infections consists of a mixed flora of aerobic, facultative anaerobic, and strictly anaerobic bacteria. This type of infection arises from bacterial colonization of the mouth.^[27–29]^

Given the risk of significant bone loss and collapse in the aesthetically sensitive anterior tooth region following tooth extraction and secondary wound healing, preservation of the vestibular alveolar bone was a priority. Additionally, augmentation using mucogingival flaps in the anterior tooth region could compromise the initial aesthetic outcome. To preserve the remaining alveolar bone around the root remnants, the chronic fistula was initially managed by crown decapitation at the gum level. The old root canal filling was subsequently revised and the canal was prepared with disinfection up to the apex. A calcium hydroxide paste (Calxyl, OCO Präparate GmbH, Germany), was placed to the canal lumen and then temporarily closed with IRM (Fig. 3C).

The goal of the root canal treatment revision was to create conditions for a complete healing of clinical and radiological symptoms, by cleaning and disinfecting the root canal.^[30]^ This allows for the resolution of apical or periapical inflammation, regardless of its extent.^[31]^

Following a 3-month healing phase of the apical periodontitis and complete closure of the fistula tracts by repeated change of medicated inserts, the fistula tracts closed completely. This allowed for the final extraction of root remnants of 11 and 21. Following clinical inspection of the periapical alveolar bone, immediate implantation was performed in regions 11 and 21 (Fig. 3D, E).

The 2-stage, minimally invasive procedure and optimized timing of extraction and implantation made it possible to exclude further extensive augmentation procedures. Further preservation of alveolar structures was ensured by immediate implantation (Fig. 3F).

**Ethical Review Statement**: Ethical review and approval were waived because the images are available for use in a scientific paper. It is a case report, and the clinical images serve to explain the evidence.

**Informed Consent Statement:** A declaration of consent for the creation of intraoral photo documentation throughout the treatment for scientific purposes was obtained verbally and in writing from all subjects involved in the study at the beginning of the treatment.

## 4. Discussion

Surgeons must carefully consider the unpredictable risks associated with granulomatous-inflammatory resorption of autologous bone grafts and the insufficient long-term stability augmented bone, particularly when planning anterior tooth implants. These factors can lead to implantological or aesthetic failure. Planning an anterior tooth implant with increased surgical interventions for additional hard and soft tissue augmentations does not typically result in the same level of aesthetic success as a minimally invasive procedure. The latter aims to preserve the natural mucogingival zone while achieving successful implantation and avoiding the need for augmentations or flap elevation.

Extensive soft tissue surgery, especially vertical releasing incisions, can result in scar formation and damage the natural mucogingival zone in the anterior tooth region. Additionally, these incisions increase the risk of recession in the adjacent natural teeth, implant crowns, and even the implant itself.^[9,11,16,17,25]^ Furthermore, preoperative infections of soft and hard tissues significantly contribute to failures, leading to early implant loss or aesthetic complications in the anterior tooth region.^[7,32]^

In contrast to the case studies presented, achieving aesthetically precise and nature-identical results in anterior tooth implantology are best accomplished through minimally invasive procedures.^[3,4,8-18]^ Extensive soft tissue procedures, such as periosteal flap elevation and vertical releasing incisions to create sliding flaps in the mucogingival region of teeth 13 to 23, should generally be avoided, as surgical reconstruction of natural mucogingival aesthetics is limited. While vertical releasing incisions, combined with sulcular incisions in the anterior tooth region (13–23 or 33–43), can be extended to the posterior premolar region, minimally invasive implantological techniques such as immediate flapless implantation are preferable. Flapless immediate implantation involves placing the implant directly into the vertically and laterally extended extraction socket immediately after tissue-sparing extraction of a noninfected, non-retainable anterior tooth.^[12-15,18]^ The implant then heals transgingivally without the need for additional vertical releasing incisions for soft tissue flap formation.

Transgingival open healing with a gingival former helps stabilize the surrounding periodontal and papillary hard and soft tissues, facilitating the creation of a natural-looking anterior implant and crown. The risk of peri-implant failure due to open healing or inadequate osseointegration resulting from distant osteogenesis between the alveolar wall and implant surface is not higher than that associated with 2-stage implantation without immediate loading.^[5,9,10,15,22,31,33-39]^ However, immediate implantation in the aesthetic anterior tooth region poses a particular challenge and should only be performed in cases that meet specific criteria.^[12-14,16,40]^

Immediate implantation should not be performed in patients with a history of chronic or acute dental infection, as bacterial contamination of the wound and implant surface can lead to peri-implant infection, premature implant loss, or aesthetic complications. Before immediate implantation, the possibility of infection at the implant site must be ruled out. If an infection is present, treatment should take precedence over implantation. Irritation-free healing and high aesthetic success can be ensured only in a noninfected implant bed, especially in the aesthetic region.

Failures in anterior tooth implantology, especially with immediate implants, often result from poor timing of extraction and implantation, or overly invasive surgical procedures. Therefore, medical indications and contraindications for immediate implantation must be carefully considered to minimize the risk of aesthetic or implantological failure.

Indications and contraindications for immediate implantation:

- Patient compliance and oral hygiene should be particularly high.
- Bone growth must be complete.
- There should be no underlying periodontal disease.
- The vestibular bony alveolus should still be completely intact.
- Any chronic or acute dental inflammation affecting the soft tissue or bone must be completely healed before implantation.
- General health risks such as blood clotting disorders, immune deficiencies, diabetes, or certain medications, serve as relative contraindications and must also be taken into account by the clinician, in terms of their individual risk, especially in the case of immediate implantation.

Clearly defining the indications and contraindications for immediate implantation in the aesthetic anterior tooth region highlights its specific and limited application. Immediate implantation is primarily considered in young adult patients, directly after an acute anterior tooth trauma with crown-root fracture, a solitary root fracture, or complete disarticulation of an anterior tooth, cases without fracture involvement of the surrounding alveolar bone, as an immediate therapeutic measure to preserve the soft tissue and bone structures.

Given the young age and sex of the patients, both with otherwise intact dentition and no systemic conditions, the primary goal was to achieve an aesthetic, natural smile. The surgical procedures employed successfully met the patients’ expectations, resulting in a high level of satisfaction for both patient and practitioner.

## Acknowledgments

The author wishes to express sincere appreciation to the 2 patients who, after undergoing years of extensive preliminary dental treatments, placed their trust in me to perform their procedures.

## Author contributions

**Conceptualization:** Thorsten Koszlat.

**Data curation:** Thorsten Koszlat.

**Funding acquisition:** Thorsten Koszlat.

**Investigation:** Thorsten Koszlat.

**Writing – original draft:** Thorsten Koszlat.

**Writing – review & editing:** Thorsten Koszlat.
